# Risk and Prognosis of Bloodstream Infections among Patients on Chronic Hemodialysis: A Population-Based Cohort Study

**DOI:** 10.1371/journal.pone.0124547

**Published:** 2015-04-24

**Authors:** Lars Skov Dalgaard, Mette Nørgaard, Bente Jespersen, Søren Jensen-Fangel, Lars Jørgen Østergaard, Henrik Carl Schønheyder, Ole Schmeltz Søgaard

**Affiliations:** 1 Department of Infectious Diseases, Aarhus University Hospital, Aarhus, Denmark; 2 Department of Clinical Epidemiology, Aarhus University Hospital, Aarhus, Denmark; 3 Department of Nephrology, Aarhus University Hospital, Aarhus, Denmark; 4 Department of Clinical Microbiology, Aalborg University Hospital, Aalborg, Denmark; 5 Department of Clinical Medicine, Aalborg University, Aalborg, Denmark; Curtin University, AUSTRALIA

## Abstract

**Background and Objectives:**

Infections are common complications among patients on chronic hemodialysis. This population-based cohort study aims to estimate risk and case fatality of bloodstream infection among chronic hemodialysis patients.

**Methods:**

In this population-based cohort study we identified residents with end-stage renal disease in Central and North Jutland, Denmark who had hemodialysis as first renal replacement therapy (hemodialysis patients) during 1995–2010. For each hemodialysis patient, we sampled 19 persons from the general population matched on age, gender, and municipality. Information on positive blood cultures was obtained from regional microbiology databases. All persons were observed from cohort entry until first episode of bloodstream infection, emigration, death, or end of hemodialysis treatment, whichever came first. Incidence-rates and incidence-rate ratios were computed and risk factors for bloodstream infection assessed by Poisson regression. Case fatality was compared by Cox regression.

**Results:**

Among 1792 hemodialysis patients and 33 618 matched population controls, we identified 461 and 1126 first episodes of bloodstream infection, respectively. Incidence rates of first episode of bloodstream infection were 13.7 (95% confidence interval (CI), 12.5–15.0) per 100 person-years among hemodialysis patients and 0.53 (95% CI, 0.50–0.56) per 100 person-years among population controls. In hemodialysis patients, the most common causative microorganisms were Staphylococcus aureus (43.8%) and Escherichia coli (12.6%). The 30-day case fatality was similar among hemodialysis patients and population controls 16% (95% CI, 13%–20%) vs. 18% (95% CI, 15%–20%).

**Conclusions:**

Hemodialysis patients have extraordinary high risk of bloodstream infection while short-term case fatality following is similar to that of population controls.

## Introduction

Renal replacement therapy (dialysis or kidney transplantation) is life sustaining in patients with end-stage renal disease (ESRD). However, factors such as disruption of natural barriers, introduction of foreign bodies in the blood stream [1], and uremic dysfunction of the immune system [2] render ESRD patients on hemodialysis (hemodialysis patients) at high risk of infectious complications.

Bloodstream infection, defined as culture-verified presence of bacteria and/or fungi in the blood, is well recognized as an important cause of morbidity and case fatality among hemodialysis patients. However, few recent studies have investigated the epidemiology of bloodstream infections [3] and recent population-based studies are lacking. The United States Renal Data System (USRDS) reported 28.2 hospitalizations with bacteremia/sepsis per 100 person-years among hemodialysis patients in 2011 [4]. This contrasts incidence rates (IRs) of bacteremia of 11.2 per 100 person-years reported by Hoen et al. in a French multicenter study [5]. Similar to the USRDS annual reports, several other studies have been based on septicemia discharge diagnoses [6, 7]. However, these diagnoses are likely to have limited sensitivity and specificity in terms of bloodstream infection [8]. A better understanding of incidence, risk factors, causative microorganisms, and outcome of bloodstream infection in hemodialysis patients could facilitate improved infection control practices and ensure appropriate empirical antibiotic treatment. Thus, updated information on these clinically important issues is highly relevant.

We therefore conducted this population-based study to delineate risk and outcome of first-time bloodstream infection among patients initiating hemodialysis for ESRD. The specific aims were to estimate incidence rates of first episodes of bloodstream infection among hemodialysis patients compared to an age- and gender-matched comparison cohort, to identify risk factors for bloodstream infection among hemodialysis patients, to describe the causative microorganisms, and to study short-term mortality following first episodes of bloodstream infection.

## Materials and Methods

### Ethics

This study was approved by the Danish Data Protection Agency (J.nr. 2013-41-152). Written consent was not required by Danish law as the study was entirely registry-based. Data were de-identified post-analysis, but prior to publication.

### Study design and setting

We conducted a population-based cohort study in Central and North Denmark Regions, from 1 January 1995 to 31 December 2010. As of 2010, the study area had a population of 1.83 million people comprising approximately 1/3 of Denmark’s population [9]. In Denmark, renal replacement therapy for ESRD is centralized to 15 specialized hospital departments of which 4 are located within the study area. Treatment for ESRD is offered free of charge by the universal tax-funded public health care system.

Since 1990, all departments treating patients for ESRD have reported data to the Danish Nephrology Registry (DNR). The DNR contains information on cause of ESRD and the complete history of renal replacement therapy; information on patients receiving temporary dialysis is not included. As of 2010, the prevalence of chronic hemodialysis patients was approximately 0.04% in Denmark [10].

The Danish National Registry of Patients (DNRP) contains information on all hospital admissions in Denmark since 1977 and all outpatient contacts since 1995 [11]. Variables include civil registration number, dates of admission and discharge, surgical procedure codes, and discharge diagnoses. Diagnoses are coded according to the International Classification of Diseases, 8th revision (ICD-8) until the end of 1993 and the 10th revision (ICD-10) thereafter. In a validation study, the DNR had a completeness of 97.2% compared to the DNRP [12].

### Study Population

Since 1968, the Danish Civil Registration System (CRS) has assigned each Danish citizen a unique 10-digit civil registration number. The CRS contains information on date of birth and death, municipality, gender, and emigration data. The civil registration number allows for accurate linkage between databases.

The DNR and the CRS were used to identify residents aged 16 years or older in the study area who were diagnosed with ESRD and received hemodialysis as first renal replacement therapy during 1 January 1995–31 December 2010. To allow for comparison of rare events, 19 persons from the general population matched on age, gender, and municipality were sampled for each hemodialysis patient through the CRS with index date defined as the date of first hemodialysis for the corresponding hemodialysis patient. These controls were born in the same year as their corresponding hemodialysis patient and resident in the same municipality (local administrative unit—level 1) at the index date. We excluded hemodialysis patients and population controls with a positive blood culture less than 14 days before index date.

### Variables

#### Definition of first episode of bloodstream infection

Bloodstream infection was defined as culture-verified presence of bacteria and/or fungi in the blood. We considered all microorganisms isolated within one day of the first positive blood culture to be part of the same episode of bloodstream infection [11]. Common skin contaminants were defined as coagulase-negative staphylococci (with exception of *Staphylococcus lugdunensis)*, *Micrococcus* species, *Bacillus* species, *Corynebacterium* species, and *Propionibacterium* species. According to Weinstein et al., two or more blood culture sets positive for one of these microorganisms had a high positive predictive value in terms of true infection [13]. Therefore, we considered common skin contaminants as part of a true bloodstream infection if *i*) the same species was isolated in two separate cultures obtained within a period of 5 days or *ii*) isolated within one day of a blood culture positive for a pathogenic microorganism. In the latter case, the bloodstream infection was categorized as polymicrobial. This algorithm is similar, but not identical to the CDC surveillance definition of primary bloodstream infections. Whereas the CDC surveillance definition of primary bloodstream infections includes clinical signs and symptoms of infections in the assessment of blood cultures positive for common skin contaminants, our definition was exclusively based on blood cultures [14]. We only considered first episode of bloodstream infection in each patient.

#### Data on bloodstream infection

Since September 1994 all positive blood cultures obtained in the North Denmark Region have been prospectively registered in the ADBakt database (Autonik AB, Ramsta, Skoldinge, Sweden). The hospital laboratory information system MADS contains dates and results of all microbiologic cultures analyzed in Central Denmark Region from March 1999 and onwards. MADS also contains data on blood cultures in the Central Denmark region analyzed during 1994–1999 with exception of two former counties (Ringkjøbing and Viborg), which were included in the study area as of 1 January 2000. In both databases, each blood culture set has a unique identification number and the patient’s civil registration number.

#### Data on comorbidity

The Charlson Comorbidity Index (CCI) score assigns a weight (1, 2, 3, or 6) to each of 19 major disease categories and is a validated measure of comorbidity [11]. In this study, we used a modified CCI score (m-CCI), which did not include renal disease, to assess the level of comorbidity based on the discharge history retrieved from the DNRP as of each person’s index date. The m-CCI score included diabetes. With the m-CCI score we defined three levels of comorbidity: low (m-CCI = 0), medium (m-CCI = 1–2), and high (m-CCI≥3).

#### Other variables

Information from the DNR was used to categorize cause of chronic renal failure as follows: Diabetes mellitus, chronic interstitial nephritis, glomerulonephritis, arterial hypertension, unspecified chronic renal failure, and other.

The date of first arteriovenous fistula surgery was identified via the DNPR (procedure codes KPBL10, KPBL10A, KPBL20, KPBL20A, KPBL30, KPBL30A, KPBL99, KPEL10, KPEL10A, KPEL10B, 87200, 87220, 87223, 87224, 87289, 87409, 87419, or 87440). We did not distinguish between native arteriovenous fistulas and arteriovenous grafts as the latter only comprised 0.26% of fistula surgery during 2000–08 [15].

### Statistical analysis

#### Follow-up

We followed each person from index date to date of first bacteremia, death, emigration from the study area, end of hemodialysis, or 31 December 2010, whichever came first.

#### Incidence rates and incidence rate ratios

We calculated IRs of first episodes of bloodstream infection overall and stratified on time since index date (<3 months, 3–11 months, and ≥12 months). Hemodialysis patients and population controls were compared by crude and adjusted incidence-rate ratios (IRRs). For adjusted IRRs we fitted a Poisson model including age, level of comorbidity, gender, and time since index date. Age (16–54, 55–64, 65–74, and ≥75 years) was included as a time dependent covariate. These age categories were similar to those used by Foley et al.[6]. However, we collapsed age categories below 55 years due to few participants.

To investigate whether the bloodstream infection IR during first year after index date changed during the study period we stratified the analysis by year of the index date (1995–99, 2000–04, 2005–09). We omitted persons with index date in 2010 from this analysis. We fitted an interval Poisson regression model with study period and time since index date (<3 months and 3–11 months) as variables and tested the assumption of no difference in IRs by Wald test.

#### Risk factors for bloodstream infection

Among hemodialysis patients the following potential risk factors were assessed: age, gender, level of comorbidity, cause of renal failure, calendar period, fistula status (no fistula, after first fistula surgery), and time since initiating hemodialysis (<3 months, 3–11 months, and ≥12 months). Age, fistula status, and time since initiating hemodialysis were included as time dependent covariates in the Poisson regression model.

#### Case fatality

We constructed Kaplan Meier plots to graph 30-day all-cause case fatality in hemodialysis patients and population controls, and used Cox regression to estimate 30-day case fatality rate-ratios (CFRR) with population controls serving as the reference group adjusting for age at first bloodstream infection (<55 years, 55–74 years, and ≥75 years), level of comorbidity, gender, and type of bloodstream infection (*Staphylococcus aureus*, *Escherichia coli*, and other).

Potential prognostic factors among hemodialysis patients were investigated using Cox regression and included: Gender, age, level of comorbidity, and type of bloodstream infection. The proportional hazard assumption was tested graphically.

Stata version 11.0 (Statacorp, College Station, Texas) was used for the analyses.

## Results

### Study population

**Fig 1:**
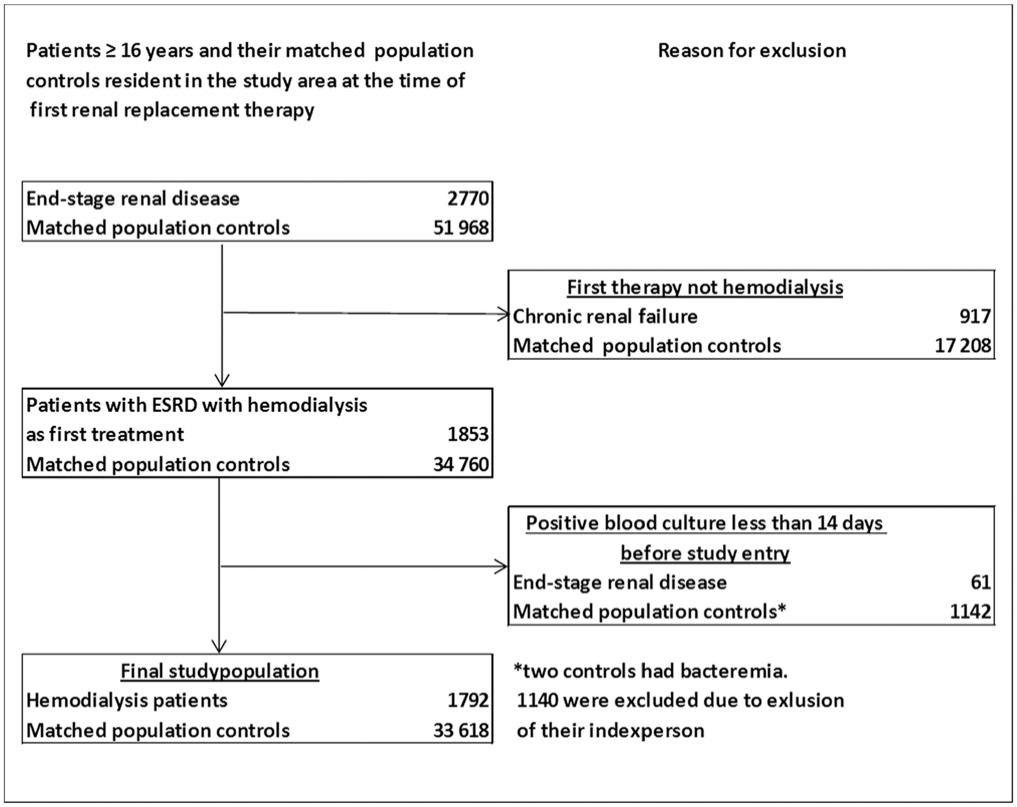
Study population. Overview.

The study population consisted of 1792 hemodialysis patients and 33 618 population controls (Fig 1 and Table 1) providing 3361 and 213 180 person-years of observation, respectively. Median follow-up was 1.13 years (interquartile range [IQR], 0.27–2.58) among hemodialysis patients and 5.80 (IQR, 2.86–9.25) years for population controls. A total of 461 and 1126 first episodes of bloodstream infection were identified in hemodialysis patients and population controls, respectively.

**Table 1:** Baseline characteristics of the study population.

	Hemodialysis		Population controls^c^	
	No.	(%)	No.	(%)
Number of participants	1792	(100.0)	33 618	(100.0)
Age at index date (years)				
16–54	421	(23.5)	7859	(23.4)
55–64	362	(20.2)	6839	(20.3)
65–74	518	(28.9)	9740	(28.9)
≥75	491	(27.4)	9180	(27.3)
Male gender	1125	(62.8)	21 075	(62.7)
Comorbidity level (m-CCI)^a^				
Low	375	(20.9)	22 397	(66.6)
Medium	656	(36.6)	8669	(25.8)
High	761	(42.5)	2552	(7.6)
Diabetes at index date^b^	561	(31.3)	2666	(7.9)
Cause of renal failure				
Unspecified chronic renal failure	520	(29.0)	-	-
Diabetes(Type I and type II)	375	(20.9)	-	-
Chronic interstitial nephritis	209	(11.7)	-	-
Arterial hypertension	161	(9.0)	-	-
Glomerulonephritis	235	(13.1)	-	-
Other	292	(16.3)	-	-
First arteriovenous fistula				
Before initiation of hemodialysis	666	(37.2)	-	-
0–30 days after first hemodialysis	294	(16.4)	-	-
>30 days after first hemodialysis	436	(24.3)	-	-
No arteriovenous fistula	396	(22.1)	-	-

Abbreviations: No. Number of participants.

^a^ Three levels of comorbidity was created based on the modified Charlson comorbidity index score (m-CCI) at the date of first treatment with renal replacement therapy “Low” (CCI = 0), “Medium” CCI 1–2, and “High” CCI > 2. See text for details.

^b^ Index date: Date of first treatment (hemodialysis) for end-stage renal disease and date of sampling for population controls.

^c^ For each patient on chronic hemodialysis, 19 persons from the background population were sampled matched on age, gender, and municipality.

### Incidence

The overall IR was 13.7 (95% confidence interval [CI], 12.5–15.0) per 100 person-years among hemodialysis patients and 0.53 (95% CI, 0.50–0.56) per 100 person-years among population controls (Table 2). In hemodialysis patients, the IR was particularly high during the first 3 months of follow-up (IR = 48.6 per 100 person-years; 95% CI, 42.1–56.1).

**Table 2:** Incidence rates of first episodes of bloodstream infection among hemodialysis patients and a matched comparison population, 1995–2010.

			Incidence Rate^c^	Unadjusted IRR	Adjusted IRR ^d^
Group	No^a^	Risk time^b^	(95% CI)	(95% CI)	(95% CI)
Overall					
Population controls	1126	213 181	0.53 (0.50–0.56)	1 (Reference)	1 (Reference)
Hemodialysis	461	3361	13.7 (12.5–15.0)	26.0 (23.3–28.9)	14.7 (13.0–16.7)
Time since study entry					
< 3 months					
Population controls	42	8363	0.50 (0.37–0.68)	1 (Reference)	1 (Reference)
Hemodialysis	187	385	48.6 (42.1–56.1)	96.7 (69.2–135)	61.5 (43.8–86.3)
3–11 months					
Population controls	132	24 544	0.54 (0.45–0.64)	1 (Reference)	1 (Reference)
Hemodialysis	98	837	11.7 (9.61–14.3)	21.8 (16.8–28.3)	13.7 (10.5–17.9)
≥ 12 months					
Population controls	952	180 274	0.53 (0.50–0.57)	1 (Reference)	1 (Reference)
Hemodialysis	176	2140	8.23 (7.10–9.53)	15.6 (13.3–18.3)	9.97 (8.41–11.8)

Abbreviations: CI, confidence interval; IRR, incidence rate ratio.

^a^ Number of first episodes of bacteremia.

^b^ Years.

^c^ Per 100 person-years.

^d^ Adjusted for age, gender, and level of comorbidity. In the overall group also adjusted for time since study entry using Poisson regression.

Over the study period, a non-significant decline in IRs of bloodstream infection within the first year of follow-up was observed in hemodialysis patients (1995–99: IR = 25.1 (95% CI, 19.7–32.1), 2000–04: IR = 23.4 (95% CI, 19.5–28.0), and 2005–09: IR = 22.6 (95% CI, 18.6–27.4) per 100 person–years). In population controls, IRs of bloodstream infection within the first year of follow-up remained stable.

### Risk factors for bloodstream infection

Table 3 presents IRs of bloodstream infection by various factors. Patients without an arteriovenous fistula had higher risk of bloodstream infection than patients with an arteriovenous fistula (IRR = 1.74, 95% CI 1.41–2.16) (Table 3). Level of comorbidity was also associated with bloodstream infection risk (medium m-CCI: IRR = 1.63, 95% CI, 1.21–2.18 and high m-CCI: IRR = 2.00, 95% CI, 1.47–2.72, compared with low m-CCI) whereas bloodstream infection risk did not vary substantially by gender, age, cause of renal failure, and year of initiation of hemodialysis.

**Table 3:** Incidence rates per 100 person-years of first episodes of bloodstream infection among hemodialysis patients by potential risk factors.

Variable	No.	Incidence rate^a^		Unadjusted IRR		Adjusted IRR^b^	
		(95% CI)			(95% CI)	(95% CI)	
Gender							
Male	292	14.0	(12.5–15.7)	1	Reference)	1	(Reference)
Female	169	13.2	(11.4–15.4)	0.94	(0.78–1.14)	0.90	(0.74–1.10)
Age group (years)							
16–54	95	13.7	(11.2–16.8)	1	(Reference)	1	(Reference)
55–64	94	14.8	(12.1–18.2)	1.08	(0.81–1.44)	1.00	(0.75–1.36)
65–74	139	14.1	(12.0–16.7)	1.03	(0.79–1.34)	0.98	(0.75–1.30)
≥75	133	12.7	(10.7–15.0)	0.92	(0.71–1.20)	0.91	(0.68–1.21)
Comorbidity (m-CCI) level^d^							
Low	72	7.81	(6.20–9.84)	1	(Reference)	1	(Reference)
Medium	161	13.5	(11.5–15.7)	1.72	(1.31–2.28)	1.63	(1.21–2.18)
High	228	18.3	(16.1–20.9)	2.35	(1.80–3.06)	2.00	(1.47–2.72)
Cause of renal failure							
Unspecified chronic renal failure^e^	123	11.9	(9.94–14.2)	1	(Reference)	1	(Reference)
Glomerulonephritis	49	11.1	(8.35–14.6)	0.93	(0.67–1.30)	0.81	(0.57–1.15)
Chronic interstitial nephritis	51	11.3	(8.55–14.8)	0.95	(0.68–1.31)	0.93	(0.66–1.31)
Other	77	15.7	(12.5–19.6)	1.32	(0.99–1.75)	1.26	(0.93–1.69)
Arterial hypertension	48	16.9	(12.7–22.4)	1.42	(1.02–1.99)	1.13	(0.80–1.61)
Diabetes type I & type II	113	17.4	(14.4–20.9)	1.46	(1.13–1.89)	1.05	(0.78–1.42)
AV fistula^f^							
After first AV fistula surgery	300	10.6	(9.47–11.9)	1	(Reference)	1	(Reference)
No AV fistula	161	28.3	(24.2–32.5)	2.85	(2.35–3.45)	1.74	(1.41–2.16)
Year of initiation of hemodialysis							
1995–99	113	13.9	(11.5–16.7)	1	(Reference)	1	(Reference)
2000–04	174	13.1	(11.3–15.2)	0.95	(0.75–1.20)	0.94	(0.74–1.19)
2005–10	144	15.1	(12.9–17.8)	1.09	(0.85–1.39)	0.79	(0.62–1.02)

Abbreviations: No., Number of first episodes of bloodstream infection; IRR incidence rate ratio; CI, confidence interval; AV fistula, arteriovenous fistula.

^a^ Per 1 00 person-years.

^b^ Adjusted for all variables in the table and time since study entry using Poisson Regression.

^c^ Calculated for the corresponding adjusted incidence rate ratios using Poisson regression.

^d^ Three levels of comorbidity was created based on the modified Charlson comorbidity index (m-CCI) score at the date of first treatment with renal replacement therapy “Low” (CCI = 0), “Medium” CCI 1–2, and “High” CCI > 2. Renal diagnosis was not included in the m-CCI score. See text for details.

^f^ A time varying covariate categorizing risk time in two categories based on the date of first arteriovenous fistula surgery.

### Microorganisms

Among hemodialysis patients *S*. *aureu*s caused 202 (43.8%) first-episodes of bloodstream infection compared with 123 (10.9%) in the comparison population (Table 4). None of the episodes in hemodialysis patients or population controls were caused by methicillin-resistant *S*. *aureu*s. *Escherichia coli* and other Enterobacteriaceae caused 58 (12.6%) and 43 (9.3%) of the episodes among hemodialysis patients, respectively. During the first three months of follow-up, *S*. *aureu*s was the causative agent in 101 of 187 (54.0%) first episodes of bloodstream infection in hemodialysis patients.

**Table 4:** Microorganisms isolated in first episodes of bloodstream infection among hemodialysis patients and matched population controls, 1995–2010.

	Hemodialysis		Population controls	
	No.	(%)	No.	(%)
Aerobic and facultative anaerobic bacteria				
Gram positive				
*Staphylococcus aureus* ^*a*^	202	(43.8)	123	(10.9)
Coagulase-negative streptococci and micrococci	31	(6.7)	31	(2.8)
Hemolytic streptococci	13	(2.8)	42	(3.7)
Non-hemolytic streptococci	9	(2.0)	38	(3.37)
Nutritional variant streptococci	2	(0.4)	1	(0.1)
*Streptococcus pneumoniae*	19	(4.1)	84	(7.5)
*Enterococcus* species	15	(3.3)	34	(3.0)
Miscellaneous gram positive microorganisms	4	(0.9)	9	(0.8)
Gram negative				
*Escherichia coli*	58	(12.6)	351	(31.2)
*Salmonella* species	2	(0.4)	6	(0.5)
Other Enterobacteriaceae	43	(9.3)	161	(14.3)
*Pseudomonas aeruginosa*	9	(2.0)	29	(2.6)
Other gram negative microorganisms	8	(1.7)	24	2.1
Yeasts and anaerobic				
Anaerobic gram positive microorganisms	4	(0.9)	3	(0.3)
Anaerobic gram negative microorganisms	9	(2.0)	25	(2.2)
Yeasts	4	(0.9)	38	(3.4)
Polymicrobial	29	(6.3)	127	(11.3)
Total	461	(100.0)	1126	(100.0)

Abbreviations: No. Number of first episodes of bloodstream infection

^a^ None of the *Staphylococcus aureus* isolates were methicillin resistant

### Mortality

**Fig 2:**
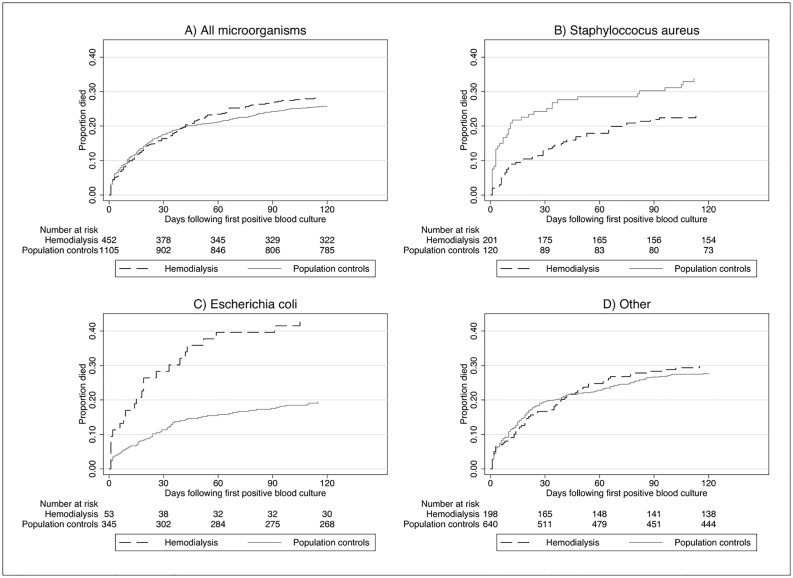
Cumulative case fatality following first episode of bloodstream infection in hemodialysis patients and a matched control population by type of microorganism.

Fig 2A–2D depicts the crude all-cause case fatality following first episode of bloodstream infection according to the isolated pathogen(s) among hemodialysis patients and population controls. Crude thirty-day case fatality from any type of bloodstream infection was 16% (95% CI, 13%–20%) and 18% (95% CI, 15%–20%) among hemodialysis patients and population controls, respectively. The crude and adjusted CFRR at 30-days were 0.93 (95% CI, 0.71–1.21) and 0.98 (95% CI, 0.71–1.35) respectively. Among hemodialysis patients, the following factors were associated with increased risk of death within 30 days: age 60–74 years (adjusted CFRR = 3.02, 95% CI, 1.45–6.30) and age>75 years (adjusted CFRR = 3.52, 95% CI, 1.657.50), compared with age<59 years and *Escherichia coli* bloodstream infection (adjusted CFRR = 1.91, 95% CI, 1.00–3.63) (Table 5).

**Table 5:** Factors associated with 30-day case fatality following a first episode of bloodstream infection among hemodialysis patients.

		Unadjusted CFRR^2^		Adjusted CFRR^3^	
Risk Factor	N	(95% CI)		(95% CI)	
Gender					
Male	292	1	(Reference)	1	(Reference)
Female	169	1.22	(0.77–1.95)	1.18	(0.74–1.88)
Age at first bloodstream infection (years)					
<59	145	1	(Reference)	1	(Reference)
60–74	183	3.45	(1.67–7.15)	3.02	(1.45–6.30)
>75	133	3.77	(1.78–7.98)	3.52	(1.65–7.50)
Comorbidity (m-CCI) level^1^					
Low	72	1	(Reference)	1	(Reference)
medium	161	2.52	(0.97–6.55)	2.21	(0.85–5.78)
High	228	2.77	(1.10–7.01)	2.47	(0.97–6.30)
Type of bacteremia					
*Staphylococcus aureus*	202	1	(Reference)	1	(Reference)
*Escherichia coli*	58	2.40	(1.27–4.54)	1.91	(1.00–3.63)
*Other*	201	1.32	(0.79–2.20)	1.16	(0.69–1.94)

Abbreviations: N, number of first episodes of bacteremia; CFRR, case fatality rate ratio; CI, confidence interval; bloodstream infection, bloodstream infection.

^1^ Three levels of comorbidity was created based on the modified Charlson comorbidity index (m-CCI) score at the date of first treatment with renal replacement therapy “Low” (CCI = 0), “Medium” CCI 1–2, and “High” CCI > 2. Renal diagnosis was not included in the m-CCI score. See text for details.

^2^ Calculated using Cox regression.

^3^ Adjusted for all variables in the table using Cox regression.

## Discussion

In this population-based study, we found that hemodialysis patients had a 26-fold higher risk of bloodstream infection compared to an age- and gender-matched background population cohort. Level of comorbidity and no arteriovenous fistula were risk factors for bloodstream infection among hemodialysis patients. *S*. *aureus* caused more than 40 percent of bloodstream infections among persons on hemodialysis which underlines the need for preventive efforts. Case fatality following the first episode of bloodstream infection was substantial highlighting the importance of clinical awareness and appropriate management of this serious condition.

To our knowledge this is the first population-based study to use clinical microbiology databases to investigate the risk of bloodstream infection among ESRD patients initiating hemodialysis. We found that the overall IR of bloodstream infection among hemodialysis patients was 13.7 per 100 person-years. In comparison, the French EPIBACDIAL study reported 51 episodes of bacteremia among 998 prevalent hemodialysis patients corresponding to an IR of 11.2 per 100 person-years [5]. The mean time on hemodialysis at study entry was 5.5 years in the EPIBACDIAL study which introduces potential for survivorship bias as long-term survivors may have a lower risk of bacteremia. In a US registry-based study on patients who had survived at least three months on hemodialysis, Foley et al. reported that IRs of first admissions for septicemia during the first year of follow-up rose from 11.6 per 100 person-years in 1991 to 17.5 per 100 person-years in 1999 [6]. A limitation here may be that the sensitivity and positive predictive value of septicemia discharge diagnoses is uncertain [8, 16]. In the HEMO study, a US multicenter trial designed to investigate effects on case fatality of dialysis dose and flow, the IR of bacteremia or sepsis was 15.9 per 100 person-years in 1846 patients[17] but included multiple events in single individuals. Overall, our results are in concordance with these older studies but contrasts with the USRDS 2013 annual report that reported 28.2 hospitalizations with sepsis/bacteremia per 100 person-years in 2011 [4]. The use of discharge diagnoses, broader definitions of clinical significance, and inclusion of multiple admissions for sepsis/bacteremia in single individuals in the USRDS report may explain the difference at least in part. In our study, establishment of an arteriovenous fistula was associated with lower risk of bloodstream infection which is consistent with numerous previous studies [1, 5, 7, 18–20]. In 2011, only 16.8% of incident US hemodialysis patients initiated treatment with an arteriovenous fistula. In our cohort, 34.8% had arteriovenous fistula surgery before initiation of hemodialysis. This difference is likely to contribute to the difference in IRs.

In the present study *S*. *aureus* caused 43.8% of bloodstream infections in hemodialysis patients. Similarly, a Canadian study on incident hemodialysis patients found that *S*. *aureus* caused 20 of 45 (44%) episodes of septicemia [19] and in the EPIBACDIAL study 39% of bacteremia episodes were caused by *S*. *aureus* [5]. In a large cohort of US dialysis patients, Abbot et al. reported that only 34% of the episodes of septicemia were caused by staphylococci [21]. However, the authors only had information on type of dialysis at treatment initiation and this information was missing in almost half of the study population. Furthermore, the study did not distinguish between S. *aureus* and coagulase-negative staphylococci which complicate comparisons with our study. Importantly, we found that *E*. *coli* was a common cause of bloodstream infection in hemodialysis patients and associated with higher case fatality compared to *S*. *aureus* bloodstream infection even when adjusting for age, and level of comorbidity. This could indicate that clinicians and hemodialysis patients are more vigilant to early signs of infections at dialysis access sites and therefore more likely to detect *S*. *aureus* bloodstream infection at an early stage. The high case fatality rate in hemodialysis patients with *E*. *coli* bloodstream infection was unexpected and may be explained by delayed diagnosis. Another reason for the high 30-day case fatality rate following E.coli BSI in hemodialysis patients may be that these infections more often than in population controls have a focus outside of the urinary tract- which may be associated with a worse prognosis. Most *S*. *aureus* infections occur in patients previously colonized with *S*. *aureus* [22] and several studies have shown decreased infection rates following nasal-or cannulation site- decolonization [23–26]. However, drug resistance have been a concern [6] and optimal clinical regimens remain to be established [27]. In a recent multicenter study, Rosenblum et al. reported that implementing and adhering to strict disinfection procedures led to a 20% reduction in rates of blood stream infections which underlines the importance of infection control practices in hemodialysis patients [28].

This study had several strengths. First and foremost, our study used population-based cohorts with high degrees of data completeness and minimal loss to follow-up. Furthermore, our estimates were not biased by multiple episodes of bloodstream infection occurring in single individuals. Finally, the use of microbiology databases allowed us to provide detailed information on microorganisms. Nevertheless, as with any clinical study, there were limitations. First, isolation of bacteria in the blood may not always represent a clinical significant finding [13, 29]. To minimize the impact of this issue on our data interpretation, we excluded common skin contaminants likely to represent contamination using a previously published algorithm essentially as described [30].Weinstein et al found that presence of two cultures positive for coagulase-negative staphylococci rarely represented contamination [29] and in a model by Tokars, presence of two cultures positive for coagulase-negative staphylococci had a high positive predictive value of clinically significant infection in patients with central vascular lines [31]. We therefore find it likely that the vast majority of episodes of bloodstream infection are real. As we only had information on date of first arteriovenous fistula surgery and lacked information on potential failure of these fistulas we could not estimate the incidence in patients with a functioning fistula. From a clinical point of view it is, however, reassuring that patients who are given an arteriovenous fistula have a lower bloodstream infection risk than patients without even when taken bloodstream infections that may be related to malfunctioning fistula into account. Finally, hemodialysis patients have a much higher risk of death than the comparison cohort which may have caused us to overestimate bloodstream infection-associated mortality. Nonetheless, as we studied 30-day case fatality the impact of this potential bias is limited due to the short follow-up period. We expect physicians to be more observant to early signs of infection among hemodialysis patients and therefore identify bloodstream infection at an earlier stage which would improve short term prognosis.

In conclusion, the risk of bloodstream infection in hemodialysis patients remains high and continues to be a cause of clinical concern. Focus on strict disinfection procedures and timely establishment of arteriovenous fistulas may reduce the risk of bloodstream infection. Further research in optimal hygienic procedures and regimens of topical antibiotic and/or use of antiseptic agents is warranted.
